# Correction: Integration of High-Resolution Physical and Genetic Map Reveals Differential Recombination Frequency between Chromosomes and the Genome Assembling Quality in Cucumber

**DOI:** 10.1371/journal.pone.0108116

**Published:** 2014-09-19

**Authors:** The PLOS ONE Staff

In the Results section there is an error in the last sentence of the first paragraph of “4. The Disparity of Recombination Frequency Along Chromosome and between Chromosomes 3 and 4” subsection. The sentence should read: Clone 4-1 and clone 4-2 markers with the same genetic position (0 cM) from the end of LG-4 were mapped at 0.7 FL and 1.9 FL cytogenetically (Figure 5 and Table 3).

In the Discussion, there is an error in the penultimate sentence of the first paragraph. The sentence should read: In addition, in the present research, the recombination suppression was also found at the end of chromosome 4, where clone 4-1 and clone 4-2 from the starting genetic positions (0 cM) were mapped onto discernible different cytogenetic position.

There are errors in Tables 2 and 3; “long arm” and “short arm” are incorrectly switched throughout both tables. Please see the correct tables here.

**Table 2:** Genetic and physical position of SSR marker-anchored cucumber chromosome 3-specific fosmid clones and satellite DNAs.

Clone Code	Clone (Fosmid) ID	Sequence position(bp)	SSR marker	Chromosome arm	cM	FL^a^	n
Ty IV	Type IV	-	-	Short arm	-	0	9
Ty I/II	Type I/II	-	-	Short arm	-	0	9
3-1	rgcfbe0_0472_B12.ab1	1121698	SSR03049	Short arm	0	6.0± 1.1	10
3-2	gcfbe0_0207_F03.ab1	10059164	SSR22514	Short arm	31	29.0± 1.9	12
3-3	gcfbd0_0441_G08.ab1	12493789	SSR13274	Short arm	40.7	37.0± 1.8	11
3-4	gcfbd0_0008_A02.ab1	16190322	SSR02244	Short arm	14.7	46.3 ±2.0	13
3-5	gcfbd0_0554_G09.ab1	16484183	CSWGATT0	Short arm	49.4	47.1 ± 1.3	9
3-6	gcfbd0_0392_E05.ab1	19248575	SSR14526	Short arm	44.399	52.5 ± 1.0	8
45S	45S rDNA	-	-	Short arm	-	55.3 ± 2.2	10
Ty III	Type III	-	-	Centromere	-	56.7 ± 1.6	9
3-7	gcfbd0_0286D12.ab1	21468111	SSR21326	Long arm	-	58.3 ± 1.3	8
3-8	gcfbd0_0643G06.ab1	23264296	SSR12032	Long arm	59.0	61.7 ± 1.6	8
3-9	gcfbd0_0073H09.ab1	25165760	SSR04632	Long arm	62.4	65.0 ± 1.8	9
3-10	gcfbe0_0072_E12.ab1	30295325	SSR05572	Long arm	84.7	83.1 ± 1.6	11
3-11	gcfbd0_0515_B12.ab1	31717595	SSR03777	Long arm	90.0	86.4 ± 2.0	6
3-12	gcfbe0_0104_H02.ab1	32147183	SSR21454	Long arm	92.4	86.8 ± 1.4	8
3-13	gcfbe0_0001_D11.ab1	38050645	SSR23517	Long arm	105.1	106.9 ± 2.4	10
Ty I/II	Type I/II	-	-	Long arm	-	112.7 ± 1.8	9
Ty IV	Type IV	-	-	Long arm	-	112.7 ± 1.8	9

a Fraction length (FL)  =  (S/T) × 37.3, where S is the distance (micrometers) from the FISH site to the end of the short arm, T is the total length of the chromosome (micrometers), and 37.3 is the length (in centimorgans) of the linkage map of chromosome 3.

**Table 3:** Genetic and physical position of SSR marker-anchored cucumber chromosome-4 specific fosmid clones and satellite DNAs.

Clone Code	Clone (Fosmid) ID	Sequence position(bp)	SSR marker	Chromosome arm	cM	FL^a^	n
Ty IV	Type IV	-	-	Short arm	-	0	10
Ty I/II	Type I/II	-	-	Short arm	-	0	10
4-1	gcfbe0_0315F11	351	-	Short arm	-	0.7 ± 0.6	8
4-2	gcfbd0_0280_E09.ab1	1301733	SSR03598	Short arm	0	1.9 ± 0.3	8
4-3	gcfbe0_0162_A08.ab1	7374241	SSR01601	Short arm	4.4	9.5 ± 1.4	9
4-4	gcfbd0_0476H06	10490315	-	Short arm	-	12.1 ± 1.8	7
Type III	Type III	-	-	Centromere	-	13.5 ± 1.6	10
45S rDNA	45S rDNA	-	-	Long arm	-	14.7 ± 0.8	10
4-5	gcfbe0_0243_E12.ab1	11480614	SSR23826	Long arm	7.5	20.0 ± 2.0	9
4-6	gcfba0_0090_G08.ab1	11633420	SSR14617	Long arm	9.3	20.7 ± 0.8	10
4-7	gcfbe0_0115_C08.ab1	12425172	SSR19565	Long arm	16.2	22.4 ± 0.6	10
4-8	gcfbe0_0098_F07.ab1	19347769	-	Long arm	-	30.2 ± 1.4	9
4-9	gcfbd0_0077_A03.ab1	20434117	-	Long arm	-	32.2 ± 1.3	8
4-10	gcfbd0_0802_E07.ab1	23135337	SSR30478	Long arm	35.1	35.9 ± 1.1	6
Type I/II	Type I/II	-	-	Long arm	-	37.3 ± 1.9	10
Ty IV	Ty IV	-	-	Long arm	-	37.3 ± 1.9	10

a Fraction length (FL)  =  (S/T) × 37.3, where S is the distance (micrometers) from the FISH site to the end of the short arm, T is the total length of the chromosome (micrometers), and 37.3 is the length (in centimorgans) of the linkage map of chromosome 4.

## References

[1] Lou Q, He Y, Cheng C, Zhang Z, Li J, et al (2013) Integration of High-Resolution Physical and Genetic Map Reveals Differential Recombination Frequency between Chromosomes and the Genome Assembling Quality in Cucumber. PLoS ONE 8(5): e62676 doi:10.1371/journal.pone.0062676 23671621 10.1371/journal.pone.0062676 PMC3646037

